# Cross-Cultural Adaptation, Validation and Reliability of the Spanish Satisfaction with Daily Occupations-Occupational Balance (SDO-OB): An Evaluation Tool for People with Mental Disorders

**DOI:** 10.3390/ijerph17238906

**Published:** 2020-11-30

**Authors:** Laura Vidaña-Moya, Mona Eklund, Jose Antonio Merchán-Baeza, Paula Peral-Gómez, Inmaculada Zango-Martín, Jenny Hultqvist

**Affiliations:** 1 Research Group GrEUIT., Escola Universitària d’Infermeria i Teràpia Ocupacional de Terrassa (EUIT), Universitat Autònoma de Barcelona, 08221 Terrassa, Spain; lauravidana@euit.fdsll.cat (L.V.-M.); indazango@euit.fdsll.cat (I.Z.-M.); 2 Department of Health Sciences, Lund University, 221 00 Lund, Sweden; mona.eklund@med.lu.se (M.E.); jenny.hultqvist@med.lu.se (J.H.); 3 Research Group on Methodology, Methods, Models and Outcomes of Health and Social Sciences (M3O), Faculty of Health Science and Welfare, University of Vic-Central University of Catalonia (UVIC-UCC), 08500 Vic, Spain; 4 Research Group InTeO, Department of Pathology and Surgery, Miguel Hernández University of Elche, 03550 Alicante, Spain; pperal@umh.es

**Keywords:** occupation, occupational balance, instrument, adaptation, validation, reliability, psychometric assessment

## Abstract

Occupation can be defined as all activities that occupy a person’s time. The Satisfaction with Daily Occupations and Occupational Balance instrument evaluates the perceived satisfaction with performance and the balance in time dedicated to different occupations. The main aim was to translate the original instrument to Spanish and examine and establish the psychometric properties. This is a quantitative, cross-sectional study conducted in two stages: translation and cultural adaptation (forward translation, expert panel, back-translation, second expert panel and pre-testing and cognitive interviewing) and collecting data to evaluate psychometric properties (homogeneity, construct validity, known-groups validity, and floor/ceiling effects). One hundred participants took part in the study, adults with a diagnosis of a mental health disorder and adults without any known health problems. The Spanish version showed known-groups validity, acceptable internal consistency, and construct validity, although the relationships with some of the indicators of discriminant validity were somewhat higher than expected. The instrument shows promise as a useful screening tool for assessing activity level and satisfaction with daily occupations among a Spanish speaking population.

## 1. Introduction

Occupation can be defined as all activities that occupy a person’s time, but as evidence has shown, the existence of a balance between personal care, productive activities, and leisure is essential for health and well-being of the person [1]. Theories and models in occupational therapy point to the human need to participate in meaningful activities, thus establishing that supporting occupational performance is a fundamental pillar of intervention within this discipline [2]. However, research has shown that subjective experiences of occupation can be more related to positive health and well-being than to the actual doing [3,4]. Subjective experiences of occupation, such as satisfaction with everyday occupations, have been shown to be important for well-being in different populations [5,6,7].

Another subjective experience of occupation that has shown to be positively associated with health and wellbeing in is occupational balance [8,9]. The definition of this term is in constant development since its first formulation as the balance between work, play, rest, and sleep [10]. Subsequently, the concept of variety has been studied, since occupational balance refers to satisfying the physical, mental, social, and rest needs, unique to everyone [11], and even the variety between active occupations and sleep or between activities that we carry out to take care of ourselves or to take care of others [12]. Later, the concept of harmonious mix appeared [13], which refers to the fact that occupations must work together harmoniously. This could refer to the individual perception of having the correct quantity and the adequate variation of occupations [14], and adding to that, performing a mixture of occupations that are significant for the person and that are aligned with their capacities and personal interests [13,15]. Finally, some authors [13,15] define occupational balance as the perceived satisfaction with performance, the balance in the time dedicated to different occupations, the achievements that are reached daily, and even the congruence between actual and desired occupations [16].

Evaluation is the first contact an occupational therapist has with a client, and it is the initial step in the clinical reasoning process [17]. This process is an integral part in the practice of occupational therapy, in which information is collected on the strengths and weaknesses of a client’s occupational performance in daily life, and involves the analysis of tasks, activities and occupations [17]. However, the use of occupation-based instruments within the discipline is limited compared to the use of disability-based instruments [18]. Moreover, changes in the client’s self-assessment of the impact of treatment can only be measured by asking the client. Subjective perceptions of occupation, such as satisfaction with everyday occupation and occupational balance, cannot be observed and reported by the occupational therapist. For this purpose, self-report instruments are needed. One of the main reasons for the lack of use of this type of scales among occupational therapists, in clinical practice as well as research, is the limited existence and accessibility of validated and reliable evaluation instruments to evaluate various aspects of participation in daily occupations [19].

The Satisfaction with Daily Occupations (SDO) is a valid and reliable assessment scale that was developed for people with mental illnesses and other diagnostic groups. It originally consisted of nine items covering the occupational areas of work, leisure activities, home management tasks, and self-care [20,21,22]. It is an easy-to-administer assessment instrument, based on an interview lasting between 10 and 20 min, that asks the person for their perceptions of their daily occupations [23]. It can be used for both detection and evaluation of results [20,21,22]. The most up-to-date version of this scale has thirteen items, and that version formed the basis for the Satisfaction with Daily Occupations and Occupational Balance (SDO-OB) instrument, which includes questions about occupational balance from a time-allocation perspective [24].

Previous studies have shown that SDO has good internal consistency in people with rheumatic and mental disorders, with Cronbach’s alpha coefficients ranging between 0.71 and 0.91 [20,21,22]. Satisfaction with daily occupations has shown to constitute a separate construct, compared to self-assessed occupational value [25], health, and quality of life [21], but unrelated with sociodemographic variables [22]. These findings indicate good construct validity, and the SDO has also shown good test-retest reliability, rs = 0.84 [20]. In addition to the satisfaction scale, the SDO also contains a level of activity scale, shown to have excellent test-retest reliability, rs = 0.92 [20]. Finally, in a study of the SDO-OB on people with mental disorders, the associations with different indicators suggested initial construct validity for the occupational balance items [24].

In the United States, 18.5% of the population is Spanish-speaking [26], which adds to the more than 400 million Spanish-speakers in the world, concentrated in South America and Spain. The American Occupational Therapy Association (AOTA) centennial vision refers to the growing diversity of the population and advocates a globally connected occupational therapy with a diverse workforce that meets the needs of society [27]. Instruments that have been validated previously are not necessarily valid in other cultural or linguistic settings [28,29]. For the SDO-OB to be used as a valid assessment in these contexts it is necessary to have a version of it that serves as a starting point. In this sense, the SDO-OB adapted to the Spanish-speaking population in the Spanish context will allow the subsequent adaptation to the rest of the Spanish-speaking contexts, covering the needs of a larger number of populations. Therefore, the main objective of this study was to conduct a cross-cultural adaptation and validation of the Spanish modified SDO-OB, Satisfacción con la Ocupación Diaria-Equilibrio Ocupacional (SOD-EO), for use by all occupational therapists who work with Spaniards.

The specific aims of this study were to i) translate the original SDO-OB to Spanish and cross-culturally adapt it to suit to the Spanish culture and ii) examine and establish the psychometric properties of the SOD-EO in terms of homogeneity and construct validity (convergent and discriminant validity), known-groups validity, and floor/ceiling effects.

## 2. Materials and Methods

The present study had a quantitative, cross-sectional design. The study was conducted in two stages: (1) translation and cultural adaptation of the SDO-OB into the SOD-EO, and (2) collecting data to evaluate the homogeneity and construct validity of the SOD-EO.

### 2.1. Translation and Cultural Adaptation

The aim of a process of translation and adaptation of an instrument is to achieve different language versions that are conceptually equivalent in each of the target countries/cultures, focusing on cross-cultural and conceptual rather than on linguistic/literal equivalence. For this purpose, WHO recommendations [30] were followed through the next steps:

Step 1. Forward translation. An occupational therapist, who is a bilingual native Spanish speaker, translated the instrument from English to Spanish focusing on conceptual translation.

Step 2. Expert panel. A panel composed of four Spanish and English-speaking occupational therapists discussed the translation process, identified discrepancies, and finalized the first draft of the Spanish version (SOD-EO).

Step 3. Back-translation. The first Spanish version of the instrument was backtranslated to English by a professional translator, whose mother tongue is English, and who had no specific knowledge in health and welfare terminology. This step allowed an identification of key concepts and problematic cultural terminology of the translation. The back-translation was reviewed by two English speaking occupational therapists.

Step 4. Second expert panel. The professional translator and the four occupational therapists of the first expert panel discussed the back-translation while also acknowledging the comments of the two English-speaking occupational therapists who reviewed the back-translation. Most of the discussions concerned terminology regarding the occupational areas in the Spanish context and establishing the cultural equivalent to some activities and health and social resources.

Step 5. Pre-testing and cognitive interviewing. To pre-test the instrument, the first version of SOD-EO was administrated to 10 persons with heterogeneity in their sociodemographic characteristics: four persons without any known health problems, four mental health services users, and two occupational therapists. After the administration, the 10 persons were interviewed regarding the comprehensibility of the instrument’s instructions and about the instrument itself. Their comments were discussed with the expert panel as a last step in the finalization of the SOD-EO.

### 2.2. Psychometric Testing of the SOD-EO

The aspects of psychometric testing investigated in this study were: homogeneity, construct validity, known-groups validity, and floor/ceiling effects. Homogeneity indicates whether items fall within a common construct. Construct validity was addressed in terms of discriminant validity, which is when two constructs show no or weak associations, and convergent validity, which refers to strong correlations between two tests that are assumed to measure the same, or very closely related, constructs [31]. Known-groups validity reflects if a measure can distinguish between two groups, known to differ on some relevant aspect. Absence of floor/ceiling effects is demonstrated when the sample’s scores are distributed on all or most scale steps, without accumulation at the lower or upper end.

### 2.3. Study Context and Participants

A total of 100 participants took part in the current study. They represented two groups of Spanish speaking adults: first, adults older than 18 years with a diagnosis of a mental health disorder who used mental health/social services (n = 50), were in an acute stage, and who had cognitive or communication difficulties. Persons who lived in an institution were excluded. The second group represented a convenience sample of adults without any known health problems (n = 50). The participants with a diagnosis of a mental disorder were recruited by the employees (occupational therapists) of the five centers that participated in the study: two community mental health centers, a center of social services for people with mental disorders, and two sheltered homes. The participants without diagnosis of mental health problems were recruited by the researchers LVM, IZM, PPG, and JAMB from the researchers’ group of friends, relatives, or co-workers, who were informed about the study by telephone or email. These researchers also matched the groups regarding gender and age based on the mental health patients’ group. Furthermore, all study participants were evaluated by LVM, IZM, PPG, and JAMB, prior to being asked to sign the informed consent.

### 2.4. Participant Characteristics

Both groups included more men than women, and age did not differ between the groups, nor did living situation. There were significant differences, however, regarding marital status, having children or not, educational level, and whether the person was currently working (Table 1).

### 2.5. Data Collection

The data were collected by means of the SOD-EO and a questionnaire concerning socio-demographic information. The data collection took place in a face-to-face interview in a secluded room in all settings. Four researchers (LVM, IZM, PPG, and JAMB) took part in collecting the data, and all of them interviewed both mental health users and healthy people. All interviewers have a research background, and two of these researchers have experience in the field of mental health, and the other two have experience in other health care fields. The data in the patient group were collected in May 2019, and the data for the group without known illness were collected in June 2019.

### 2.6. Sociodemographic and Clinical Data

A questionnaire addressing socio-demographic and clinical data was devised specifically for the present study. The participants answered questions about gender, age, marital status, living situation, having children, educational level, and currently working or not. There was also a question in the group for the mental health/social service user group addressing whether the participant had a medical diagnosis. Those who had were then asked to report that diagnosis. The referent professionals were asked to confirm the diagnosis of the participants based on medical history recorded in their centers.

### 2.7. Questions to Evaluate Construct Validity

The SOD-EO is an interview-based questionnaire that assesses the person’s satisfaction with their daily occupations and their perception of balance within four different domains of daily life: work, leisure, home chores, and self-care [24,32]. The 4 domains comprise in total 13 items. For every domain there is an occupational balance item that addresses the allocation-of-time facet of occupational balance. There are also two additional items that concern general satisfaction with daily occupations and general occupational balance.

The 13 items of the 4 domains are rated in two ways; first, the participant states whether he/she presently performs daily occupations within the domain. The number of activities in which the participant is engaged reflects the level of activity score, which may thus range between 0 and13. The second rating indicates satisfaction with daily occupations, where the participant rates each item ranging from 1 (could not be worse) to 7 (could not be better). The participant is instructed to rate his/her satisfaction regardless of being currently engaged or not engaged in the occupation addressed in the item. The satisfaction score may range between 13 and 91. The question concerning satisfaction with everyday occupations in general was rated on a five-point Likert scale where 1 = very dissatisfied and 5 = very satisfied. Regarding the balance items, there are five response alternatives: way too little (−2), too little (−1), just enough (0), too much (1), and way too much (2). The balance ratings were not summarized but analyzed separately.

The summed satisfaction score, the activity level score of the SOD-EO, and the single questions regarding general occupational balance were used for the evaluation of construct validity of the SOD-EO. The association of each of these variables with the item reflecting general satisfaction was analyzed to investigate convergent validity, thus as the indicator variable. It was hypothesized that the SOD-EO summed satisfaction scale would show a strong correlation with the indicator variable since they are supposed to assess the same type of phenomenon. Activity level and occupational balance were hypothesized to show moderate relationships with the single satisfaction item, given that the constructs are similar as they address subjective perceptions of occupation, but also different since they reflect varying subjective aspects [32].

To address discriminant validity, a construct that differed from all of these was needed, and we chose to include an assessment of self-rated health, namely the first item from the MOS short form-36 (SF-36) [33]. The question is worded “how would you say your health is in general” and is rated on a five-point rating scale where 1 = excellent and 5 = poor. This single item measure has been found to be a valid broad summary rating of overall subjective health [34]. Regarding discriminant validity, it was hypothesized that the summed satisfaction with everyday occupations score, activity level, and general occupational balance would all show a weak to moderate correlation with self-rated health. The rationale behind this assumption was that health as a phenomenon is different from perceptions of occupation, but still often significantly related, as shown in previous research [20].

### 2.8. Ethical Considerations

Ethical approval was obtained for the study from Ethics Committee of the University of Vic (56/2018). All participants provided written, informed consent. The personal data of the participants were protected according to the Organic Law of Protection of Personal Data 03/12/18. This research was governed by the ethical principles reflected in the Declaration of Helsinki.

### 2.9. Data Analyses

Differences between the groups regarding socio-demographic variables were explored by applying the independent samples’ t-test and the Chi-square test. Cronbach’s alpha was used to assess internal consistency. Spearman correlations, based on the raw scores, were employed to calculate associations between ordinal variables. As suggested by Streiner and Norman [31], an alpha value between 0.70 and 0.90 and an item-total correlation between 0.20 and 0.80 were considered acceptable cut-offs. Since the data were mainly based on ordinal scales, non-parametric tests were used for further statistical analyses. Group comparisons regarding occupational satisfaction and activity level were analyzed by means of the Mann-Whitney U-test and the Chi-square test. The raw score of general occupational balance was analyzed by means of the Mann-Whitney U-test to investigate difference between the groups. In a second step, the participants were grouped, based on raw scores regarding general occupational balance, into “under-occupied” (score of −2 or −1), “in balance” (score of 0), and “over-occupied” (score of 1 or 2), and the Kruskal-Wallis test was applied to analyze whether this grouping was of relevance to self-rated health. Differences between the groups were also calculated in terms of effect size (Cohen’s *d*). The floor/ceiling effect was explored by the distribution of frequencies regarding both activity level and summed satisfaction with daily occupations. If fewer than 15% of the respondents achieved the lowest or highest scores, respectively, it was considered as indicating an absence of floor/ceiling effects [35].

The psychometric analyses and statistical methods of this study are presented in Table 2. In accordance with Cohen (1988), a correlation coefficient <0.3 was a weak correlation, 0.3–0.5 moderate, and >0.5 a strong correlation [36]. The data analyses were performed with the IBM-SPSS software, version 26 (IBM, Chicago, IL, USA). The level of significance was set at <0.05.

## 3. Results

### 3.1. Internal Consistency

The Cronbach’s alpha for the present sample was 0.751 (95% C.I. 0.673—0.818). The corrected item total correlation ranged between 0.281 and 0.588 with a mean of 0.375. Table 3 shows the Corrected Item–Total correlation and Cronbach’s alpha if item deleted.

### 3.2. Known-Groups Validity

The discriminating ability of the SOD-EO was evaluated by comparing the two samples. The result of the analysis comparing the summed satisfaction with daily occupations score showed that the healthy group scored significantly higher (*p* = 0.005; Cohen’s *d* = 0.515, 95% C.I. 0.117–0.913) than the group of mental health/social services users. The analysis regarding activity level showed a significant difference between the groups (chi2 = 22.7; df = 10; *p* =0.012; Cohen’s *d* =0.684, 95% C.I. 0.281–1.087), likewise the healthy group scored significantly higher. The Mann-Whitney U-test was applied to compare general balance between the two groups and the result was shown to be insignificant (*p* = 0.223; Cohen’s *d* = 0.204, 95% C.I. −0.189–0.597). Descriptive statistics are shown in Table 4.

### 3.3. Construct Validity

#### 3.3.1. Convergent Validity

The SOD-EO summed satisfaction scale and activity level were strongly related (r_s_ = 0.521, *p* < 0.001). The correlation between summed satisfaction and general satisfaction was moderate at 0.399 (*p* < 0.001). Finally, the correlation between the general satisfaction scale and general occupational balance was moderate (r_s_ = 0.375, *p* < 0.001).

#### 3.3.2. Discriminant Validity

The correlation between SOD-EO summed satisfaction and self-rated health was moderate at 0.332 (*p* = 0.001). Moreover, the correlation between SOD-EO activity level and self-rated health was weak at −0.258 (*p* = 0.010).

The correlation between general occupational balance scores and self-rated health was weak at −0.215 (*p* = 0.032). We also used the Kruskal-Wallis test to analyze whether the grouping into under-occupied, in balance, and over-occupied was of relevance to self-rated health and found a *p*-value of 0.230.

### 3.4. Floor and Ceiling Effects

The analyses of the distribution of frequencies regarding summed satisfaction with daily occupations and activity level showed that less than 15% of the respondents achieved the lowest or the highest scores which indicates that there were no floor or ceiling effects.

## 4. Discussion

This study investigated the psychometric properties of the SOD-EO, a translated and culturally adapted version of the SDO-OB aimed for use within Spaniards. The SOD-EO satisfaction scale showed an acceptable internal consistency, as indicated by a Cronbach’s alpha at 0.751 and all corrected item total correlations (except for one item; satisfaction with housework = 0.281) being >0.30 [35]. The activity level score is only based on the number of activities the person is engaged in and calculating internal consistency is therefore not relevant.

Furthermore, the results showed that the SOD-EO activity level and satisfaction scale had the ability to discriminate between service users with mental health problems and adults without any health problems, thus demonstrating known-groups validity. As expected, participants without health problems reported significantly higher activity levels and satisfaction scores than the group with mental health problems. These findings are in line with previous research on the SDO’s discriminating ability when comparing a healthy sample with a sample with illness/disability [23,37]. General occupational balance, however, did not discriminate between service users with mental health problems and adults without any health problems. The SDO-OB/SOD-EO [24] builds upon the most recent version of the SDO and uses the same 13 items, but also includes additional questions about occupational balance. Previous research on the SDO-OB targeted people with mental illness specifically, i.e., there was no comparison with another group [24]. Accordingly, this is the first study of SDO-OB that uses the general occupational balance item to investigate known-groups validity, and future studies can explore this further.

Regarding construct validity in terms of convergent validity, SOD-EO satisfaction and activity level were strongly related (*p* = 0.521), which is in line with other studies of SDO [38]. The correlation between the summed satisfaction score and general satisfaction was also significant, although in the realm of moderate (*p* = 0.399), which is weaker than what other studies have reported [38,39].

Regarding discriminant validity the correlation between SOD-EO summed satisfaction and self-rated health showed to be moderate at −0.332, and that between the SOD-EO activity level score and self-rated health was −0.258. This is similar to previous research in the SDO [20]. However, in the present study’s sample of persons with mental health problems, neither over-occupation nor under-occupation were associated with worse health, which deviates from previous research reporting worse self-rated health among people with mental disorders who saw themselves as under-occupied [24]. The analyses addressing construct validity thus yielded somewhat inconsistent results. These results taken together suggest that the construct validity of the SOD-EO in terms of discriminant validity should be investigated further in future research.

Finally, the results showed that there were no floor or ceiling effects concerning summed satisfaction scores or activity level.

Achieving the validation of a new assessment tool focused on occupation in the context of Spanish occupational therapy will facilitate focusing efforts beyond the improvement of abilities and address opportunities to achieve the well-being of people through occupational participation [40]. People with mental health disorders tend to spend a lot of time passively, doing solitary activities, and spending less time at work and other productive activities, experiencing a clear risk of being underoccupied [41,42]. Moreover, disruptions or restrictions in activity engagement can negatively impact health and well-being [43]. Therefore, improving this situation of occupational imbalance becomes one of the aims of treatment in occupational therapy [44]. It is for this reason that SOD-EO can become a valuable tool to identify people’s occupational needs from a subjective point of view.

The use of self-reported outcome instruments in health research has become increasingly important because the perspective of therapy users is an essential part of evaluating the efficacy of a treatment [45]. To determine the most effective occupational therapy interventions, larger-scale studies are needed, including consistent outcome measures related to occupational aspects as well as physical and mental health outcomes [43]. Since occupational balance guides much of the clinical practice of occupational therapists [45] fidelity measure that ensures consistency in the evaluation of interventions in occupational therapy in Spain.

### Strengths and Limitations

The process of translation and adaptation of SDO-OB into SOD-EO followed the guidelines from WHO [30] and used forward-translations and back-translations incorporating the perspective from both professionals and people with mental health problems. The study was not based on systematic sampling. Convenience sampling, which is common in psychometric pilot studies such as the current one, limits the generalizability of the findings. The study was the first to investigate the Spanish version SOD-EO, however, and the aim was to study initial psychometric properties. The study sample consisted of 100 participants. We propose that construct validity should be addressed further in future research with a larger sample of persons with mental disorders and persons without mental disorders. This would add information about the properties of SOD-EO. There is no such thing as fixed properties of an instrument, however, and they will always vary somewhat with the sample. In the assessment of construct validity, single items (overall occupational satisfaction, general occupational balance, and overall health) were used, which may be regarded a weakness. There is, however, research supporting the use of single-item questions [34]. Given these limitations, we regard the findings concerning the psychometric properties of SOD-EO as promising but preliminary.

After its translation and validation, we recommend that this instrument be used and applied in the clinical and research context of Spain, due to the variability that exists in the language and the culture throughout the Spanish speaking countries [46,47]. Spanish is the second largest mother tongue in the world in regard to number of speakers and it is the official language in 21 countries, spoken today by over 577 million people as a native tongue, second language, or foreign language [48]. For these reasons a more extensive validation of all Spanish speakers in the world has not been possible in the present study. We propose that the translation, cultural adaptation, and psychometric testing of the SOD-EO in the present study can serve as a starting point for the use of the SOD-EO as an evaluation tool for people with mental disorders in Spanish speaking populations.

## 5. Conclusions

The SOD-EO showed known-groups validity, acceptable internal consistency, and construct validity, although the relationships with some of the indicators of discriminant validity were slightly higher than expected. Construct validity of the SOD-EO in terms of discriminant validity should be investigated further in future research. We propose that the SOD-EO shows promise as a useful screening tool for assessing activity level and satisfaction with daily occupations for clinicians and researchers in Spain. The SOD-EO can complement instruments addressing other aspects of occupation such as occupational performance. Future studies of the SOD-EO should address other client groups and investigate sensitivity to change, which is of importance for measuring outcomes in practice and in research. In addition, further validation and research are warranted for the use of SOD-EO in other Spanish speaking countries. 

The SOD-EO can be retrieved from the corresponding author by request.

## Figures and Tables

**Table 1:** Socio-demographics and clinical characteristics of the participants.

Variable	Group 1 (Mental Health Group)	Group 2 (Healthy Group)	Group Comparisons
Sex, male, n (%)	31 (62.0)	33 (66.0)	ns.
Age, mean (std deviation)	44.4 (9.7)	44.7 (10.8)	ns.
Marital status:			
Single, n (%)	35 (70.0)	16 (32.0)	*p* = 0.004
Married/cohabitant, n (%)	7 (14.0)	29 (58.0)	
Divorced, n (%)	7 (14.0)	3 (6.0)	
Widowed, n (%)	1 (2.0)	2 (4.0)	
Living situation ¹:			
Own home, n (%)	32 (65.3)	32 (64.0)	ns.
Rental property, n (%)	17 (34.7)	18 (36.0)	
Having children, yes n (%)	10 (20.0)	29 (58.0)	*p* < 0.001
Number of children, mean (min-max)	0.68 (1–9)	1.08 (1–4)	ns.
Educational level:			
No studies, n (%)	2 (4.0)	2 (4.0)	*p* < 0.001
Elementary school, n (%)	12 (24.0)	3 (6.0)	
Secondary school, n (%)	28 (56.0)	17 (34.0)	
University degree, n (%)	8 (16.0)	28 (56.0)	
Currently working, yes n (%)	5 (10.0)	45 (90.0)	*p* < 0.001
Diagnosis: n (%)			
Psychosis	23 (46)	n.a.	n.a.
Affective disorders	16 (32)		
Other	11 (22)		

Note: ¹ 1 missing value in group 1. ns. = non-significant. n.a. = non-applicable.

**Table 2:** Psychometric analyses, data, and statistical methods.

Psychometrics	Data	Methods of Analyses
Internal consistency	The satisfaction scale of the SOD-EO.	Cronbach’s alpha and corrected item-total correlations.
Known-groups validity	The satisfaction scale, the level of activity scale of the SOD-EO, and general occupational balance.	Inferential statistics, comparison between the two groups. The Chi-Square Test and the Mann-Whitney U-test.
Construct validity	Convergent validity Summed satisfaction with everyday occupations and activity level. Summed satisfaction and general satisfaction with everyday occupations. General satisfaction and general occupational balance.	Correlations between summed satisfaction with everyday occupations and activity level; and summed satisfaction and general satisfaction with everyday occupations; and general satisfaction with everyday occupations and general occupational balance. Spearman’s Rank Correlation. Associations between the SOD-EO summed satisfaction scale and self-rated health, activity level and self-rated health, general occupational balance, and self-rated health. Spearman’s Rank Correlation. Kruskal-Wallis Test.
Floor and ceiling effect	The satisfaction scale and the level of activity scale of the SOD-EO.	Descriptive statistics, distribution of frequencies.

**Table 3:** Corrected Item–Total correlation and alpha value if item deleted.

Item	Corrected Item–Total Correlation	Cronbach’s Alpha If Item Deleted
Satisfaction with work	0.326	0.742
Satisfaction working the last two months	0.361	0.738
Satisfaction with rehabilitation	0.419	0.731
Satisfaction with organized hobby	0.321	0.742
Satisfaction with hobby on independent basis	0.588	0.711
Satisfaction with cultural activities	0.314	0.742
Satisfaction with housework	0.281	0.746
Satisfaction with repair or maintenance jobs	0.386	0.735
Satisfaction with planning and organizing of the housework	0.332	0.740
Satisfaction with care of children, parents, or significant others	0.335	0.740
Satisfaction with daily self-care	0.393	0.736
Satisfaction with physical exercise	0.439	0.728
Satisfaction with rest, relaxation, or recharging their batteries	0.383	0.735

**Table 4:** Descriptive statistics of scores for satisfaction with everyday occupations scores, activity level and general occupational balance.

Variable	Group 1 (Mental Health Group)	Group 2 (Healthy Group)
Satisfaction with everyday	67.5 (41–88)	74 (40–88) *
occupations, median (min-max)		
Activity level, median (min-max)	8 (4–13)	11 (4–13) **
General occupational balance:		
Under-occupied n (%)	23 (46)	17 (34)
In balance n (%)	11 (22)	9 (18)
Over-occupied n (%)	16 (32)	24 (48)

Note: * *p* < 0.05, ** *p* < 0.01.
